# Trace Elements and Viral Infectious Diseases: Dual Roles in Pathogenesis and Immunity

**DOI:** 10.3390/idr18020022

**Published:** 2026-03-10

**Authors:** Carla Mariana da Silva Medeiros, Michely da Silva Sousa, Lucas Hestevan Malta Alfredo, Jemmyson Romário de Jesus, Cícero Alves Lopes Júnior

**Affiliations:** 1 Grupo de Estudo em Bioanalítica (GEBIO), Department of Chemistry, Federal University of Piauí–UFPI, Teresina 64049-550, Piauí, Brazil; carlamarianadasilvamedeiros@gmail.com (C.M.d.S.M.); michelysousacx@gmail.com (M.d.S.S.); 2 National Institute of Science and Technology of Bioanalytics Lauro Kubota (INCTBio-LK), Institute of Chemistry, Universidade Estadual de Campinas (UNICAMP), Campinas 13083-970, São Paulo, Brazil; lucas.alfredo@ufv.br; 3 Research Laboratory in Bionanomaterials, LPbio, Department of Chemistry, Federal University of Viçosa, Viçosa 36570-900, Minas Gerais, Brazil

**Keywords:** viral infection, trace elements, immune response, oxidative stresses, human health

## Abstract

Introduction: Trace elements such as zinc, selenium, iron, copper, and manganese play a vital role in human health—especially in how the immune system responds and how the body handles viral infections. These trace elements have complex and sometimes context-dependent effects: while they can strengthen the body’s defenses, imbalances may promote viral replication and worsen tissue damage. Methods: Relevant articles discussed in this narrative review were identified through searches in major databases, including PubMed, Scopus, and Web of Science, primarily those published from 2020 onwards. Discussion: In this review, we examine key findings on how trace elements influence antioxidant defense, modulate viral replication, and regulate cytokine signaling, considering the context of innate immunity and the pathology of viral diseases. We discuss their impact on major infections such as HIV, viral hepatitis, and coronaviruses, highlighting how deficiencies or excesses of certain minerals can affect disease severity, immune responses, and clinical outcomes. The therapeutic use of trace element supplementation is also examined, emphasizing the importance of maintaining proper balance to avoid harmful effects. Conclusions: These findings contribute to a deeper understanding of the complex relationship between micronutrients and viral infections, which can inform the development of more effective prevention and treatment strategies. This review underscores the need for further clinical and experimental studies to define optimal levels of these elements in different health and disease scenarios.

## 1. Introduction

Viruses are infectious agents composed of a single type of nucleic acid (RNA or DNA) enclosed within a protein capsid, which may be surrounded by a lipid envelope [1]. Although not the smallest infectious entities, since viroids and prions are smaller, viruses are among the leading causes of human disease, with more than 300 species recognized as pathogenic to humans [2].

Human viral pathogens comprise a broad and diverse group, causing acute and chronic diseases that range from mild infections to severe syndromes. Representative examples of viruses that affect humans include: (*i*) retroviruses (human immunodeficiency virus type 1, HIV-1) [3], (*ii*) hepatotropic viruses (including hepatitis B virus, HBV, and hepatitis C virus, HCV) [4], (*iii*) herpesviruses (herpes simplex virus, HSV) [5], (*iv*) respiratory viruses (influenza viruses) [6] and coronaviruses [7], (*v*) oncogenic viruses (human papillomavirus, HPV [8], (*vi*) enteric viruses (rotavirus) [9], (*vii*) exanthematous viruses (rubella virus) [10], and finally (*viii*) filoviruses (Ebola virus) [11].

**Figure 1:**
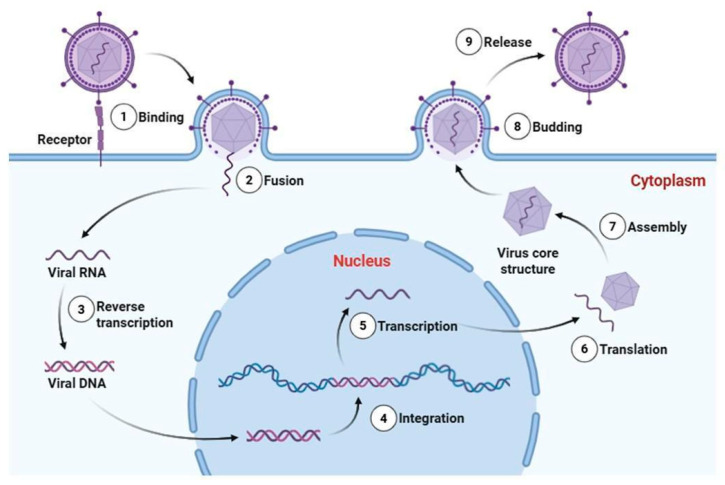
Schematic representation of the replication cycle of an enveloped retrovirus (Baltimore Group VI). The infection process includes (1) receptor-mediated binding, (2) membrane fusion and entry, (3) reverse transcription of the single-stranded RNA genome into double-stranded viral DNA, (4) integration of proviral DNA into the host genome, (5) transcription by host RNA polymerase II, (6) translation of viral proteins in the cytoplasm, (7) assembly of viral RNA and structural proteins into core particles, (8) budding from the plasma membrane, and (9) release of mature virions. Essential trace metals such as Zn, Fe, Cu, and Se can modulate multiple stages of viral pathogenesis, influencing viral enzyme activity, redox balance during replication, structural stability of viral proteins, and host immune responses. Viral RNA, proviral DNA, and structural proteins are represented as distinct molecular entities. This retroviral model is presented to illustrate defined molecular stages of viral infection; reverse transcription and genome integration are not universal features of all RNA viruses. Created in Biorender (https://BioRender.com).

The viral life cycle begins with entry into the host, followed by attachment to susceptible cells, penetration, replication, and subsequent cell damage that can culminate in cell death [12]. Figure 1 presents a schematic representation of the replication cycle of an enveloped retrovirus, illustrating the main molecular stages of viral infection, including reverse transcription and genome integration. At any stage of this process, viral propagation can be interrupted by host defense mechanisms, primarily through innate and adaptive immune responses [1].

Viral infection is driven by a complex interaction between viral factors, such as genetic variability, replication dynamics, immune evasion mechanisms, and host-related factors, including nutritional status, metabolic balance, and immune competence [13]. Among these host factors, trace elements have emerged as essential modulators [1,14]. They function as indispensable cofactors for a wide range of enzymes, transcriptional regulators, and immune signaling pathways. However, they can also be utilized by viruses to increase replication efficiency and promote persistence in the host [1,14].

Elements such as copper (Cu), iron (Fe), manganese (Mn), selenium (Se), and zinc (Zn) are essential for maintaining immune competence and redox balance [15]. However, both deficiency and overload can compromise host defenses, impair antiviral mechanisms, or create conditions favorable to viral proliferation [16]. For instance, Fe imbalance can influence viral replication (depending on the virus), while Zn and Se deficiencies are associated with impaired interferon responses and increased viral pathogenesis [17,18,19]. This dual role highlights the complexity of host–virus interaction mediated by trace elements. In other words, sufficient micronutrient levels enhance antiviral immunity and limit viral replication. However, specific viruses have developed strategies to manipulate the host’s trace element metabolism to their advantage [1]. In this context, it becomes crucial to clarify the precise roles of trace elements in viral infections.

In this review, we critically evaluate the roles of trace elements in viral pathogenesis and host immunity, highlighting their dual nature as protective factors and facilitators of infection. Furthermore, we provide a comprehensive overview of how trace element homeostasis influences the trajectory of viral infections and identify strategies for therapeutic and nutritional interventions.

## 2. Trace Elements in Human Physiology

Although some chemical elements are required in trace amounts, they play disproportionately critical roles in maintaining human physiological balance [20]. These elements are not randomly present in tissues and fluids but are integral components of complex biochemical systems that regulate cellular function, enzymatic activity, and metabolic homeostasis [21]. Their physiological importance is attributed not only to their presence but also to their bioavailability, chemical speciation, and dynamic interaction with ligands and macromolecules [1,21].

In the human body, chemical elements can exist both free and bound to biomolecules, such as amino acids (e.g., glutamate, aspartate), small organic acids (e.g., picolinate, citrate, ascorbate), or proteins [22]. This binding is responsible for the stability, solubility, transport, and cellular absorption of metals. Any imbalance in metal–ligand homeostasis, whether due to excessive complex formation, ligand depletion, or metal overload, can lead to pathological changes, oxidative stress, or disruption of enzymatic cascades [23]. Based on their abundance in biological systems, chemical elements can be categorized into three important groups: (i) macroelements, (ii) trace elements, and (iii) ultra-trace elements [24].

While macroelements such as calcium (Ca), potassium (K), and sodium (Na) are required in gram quantities and primarily perform structural and electrochemical functions [25], trace and ultratrace elements function predominantly as catalytic and regulatory agents, often in concentrations ranging from micrograms to milligrams per kilogram of tissue [24]. Among trace elements, Fe is a main example of a dual-function element, essential for oxygen transport and mitochondrial respiration, but potentially toxic in its free form due to its ability to catalyze the formation of reactive oxygen species (ROS) [26]. Similarly, Zn is essential for the activity of more than 300 enzymes and transcription factors, but its deficiency or excess can seriously impair immune and neurological functions [27]. Copper participates in redox reactions, but its inadequate management is implicated in neurodegenerative diseases such as Wilson’s disease and Alzheimer’s disease [15]. Ultra-trace elements, such as selenium (Se), cobalt (Co), chromium (Cr), and iodine (I), are involved in critical hormonal and antioxidant pathways [24]. For example, Se is a component of selenoproteins such as glutathione peroxidase, which protects cells against oxidative damage, while I is essential for the biosynthesis of thyroid hormones [28].

It is important to emphasize that the biological activity of trace elements is influenced by dosage and chemical form [29]. All essential trace elements have a narrow therapeutic window. In other words, their benefits are limited within specific concentration ranges. Above this range, they can be toxic [29]. This is particularly evident for elements such as Se and Cr, where deviations from the optimal range can result in cytotoxicity or systemic toxicity [30]. In this context, even at low concentrations, trace elements significantly influence essential physiological systems, including endocrine regulation, immune response, and neurodevelopment [14].

The dose–response relationship of trace elements typically follows a U-shaped curve, with both deficiency and excess leading to functional impairments [31]. Furthermore, interactions between trace elements can be synergistic or antagonistic. For example, high dietary Zn intake can inhibit Cu absorption, while Fe and Mn compete for similar transport pathways [32]. Therefore, understanding elemental interactions is vital for both clinical nutrition and the development of therapeutic strategies.

In general, this information has significant implications for understanding the role of trace elements in modulating viral infections and the host immune response. For instance, Chaubey et al. [33] demonstrated that elevated intracellular Fe levels increase the severity of COVID-19 infection. In this study, cultured cells with induced Fe overload were assessed for their susceptibility to infection with a pseudovirus engineered to express the SARS-CoV-2 spike protein, along with analysis of the resulting inflammatory responses. The results revealed that excess Fe significantly increased the expression of the ACE2 receptor in host cells, thus increasing the ability of the pseudovirus to infect these cells. Furthermore, Fe-overloaded cells exhibited a synergistic increase in markers of oxidative and nitrosative stress, specifically ROS and reactive nitrogen species (RNS). Following stimulation with the spike protein, there was also a marked increase in the production of pro-inflammatory cytokines, including IL-1β, IL-6, IL-8, and TNF-α, as well as chemokines such as CXCL1 and CCL4. These in vitro findings were corroborated by experiments in an animal model, reinforcing the pro-inflammatory role of Fe in the context of viral infection.

In another study conducted by Leal et al. [34], the urinary excretion profiles of multiple metals, including Ca, Co, Cu, Fe, Mg, Ni, Se, and Zn, were quantitatively analyzed in individuals diagnosed with COVID-19 (*n* = 35) and compared to a healthy control group (*n* = 60). The results demonstrated a significant increase in the urinary concentrations of all metals analyzed in the COVID-19 group compared to the control group, suggesting an alteration in systemic metal homeostasis potentially associated with the pathophysiology of SARS-CoV-2 infection. Interestingly, Cu was the only element found to be significantly reduced in the urine of COVID-19 patients, indicating a possible alteration in the metabolism or redistribution of this metal during the course of the infection. Elevated levels of other metals in urine may reflect increased oxidative stress, renal dysfunction, or inflammatory responses triggered by viral infection, which can disrupt trace element regulation and promote abnormal excretion patterns [34].

These findings highlight the importance of monitoring trace elements and essential elements during infectious diseases, particularly in the context of viral infections, where metal dysregulation may contribute to disease severity or progression.

## 3. Metal Imbalance and Susceptibility to Infections

**Figure 2:**
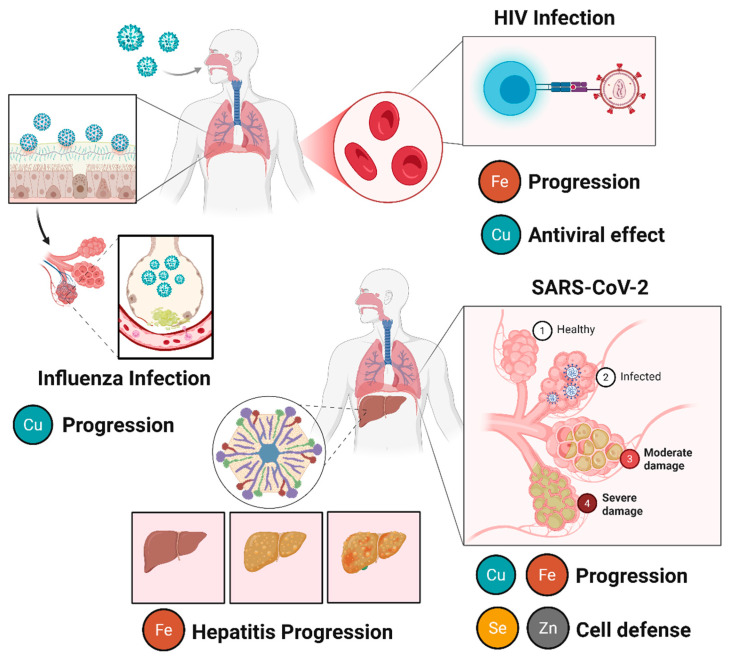
The influence of trace element immunosuppression on viral diseases. In HIV infection, iron (Fe) promotes viral replication and disease progression, whereas copper (Cu) exhibits antiviral properties, contributing to virus inhibition via cuproptosis-induced cell death. In influenza infection, copper is associated with antiviral activity and modulating immune response, acting as a protective factor. In viral hepatitis progression, Fe accumulation is related to increased oxidative stress, liver damage, and disease worsening. In SARS-CoV-2 (COVID-19) infection, disease progression is influenced by multiple metals. Iron (Fe) and copper (Cu) are associated with inflammatory processes and tissue damage, while zinc (Zn) and selenium (Se) play protective roles by contributing to antiviral immunity, redox homeostasis, and cellular defense mechanisms. Created in Biorender (https://BioRender.com).

Several trace elements play essential roles in viral infections by contributing to the survival and replication of viruses through their participation in metalloproteins involved in viral binding and entry into host cells. They also modulate the host’s immune response [35,36,37]. Dysregulation of metal homeostasis during infection can directly influence the characterisation and progression of viral diseases by affecting the oxidative and inflammatory mechanisms associated with pathogenesis [1]. Some studies have associated trace element deficiency or overload with viral infectious diseases, including SARS-CoV-2, HIV, hepatitis B and C, and influenza (Figure 2). Therefore, maintaining the balance of metals in the body through proper nutrition and supplementation, when necessary, is essential for proper bodily function and an effective immune response. Thus, this section summarizes the benefits of some metals and the consequences of deregulating their homeostasis for physiological processes.

### 3.1. Role of Copper and the Immune System

Copper is an essential trace element for human health, which is present in almost all living organisms. This metal plays a role mainly in biological functions, including as a key catalytic cofactor associated with various cellular enzymes, including bacterial electron transfer reactions, as well as p-hydroxyphenyl pyruvate hydrolase, tyrosinase, cytochrome c oxidase, lysyl oxidase (LOX), NADH dehydrogenase, dopamine beta-hydroxylase, and copper–zinc superoxide dismutase (Cu-Zn SOD) [38,39]. In addition, Cu plays a role in crucial cellular processes such as mitochondrial energy transformation, neurotransmitter synthesis, cell signaling, protection against oxidative stress, and Fe transport [40]. Therefore, Cu concentrations in the body must be maintained within the Recommended Dietary Allowance (RDA) of a daily dose of 0.9 mg/day and an upper limit (UL) of no more than 10 mg/day [41].

The human body absorbs Cu predominantly in the small intestine, particularly in the duodenum and proximal jejunum, via specific transporters in the enterocyte membrane [38]. During this process, Cu molecules in food are reduced by reductases in the apical membrane from the cupric form (Cu^2+^) to the cuprous form (Cu^+^) by the Ctr1 transporter (Copper Transporter 1) and, to a lesser extent, DMT1 (Divalent Metal Transporter 1) [42]. Subsequently, upon reaching the liver, this mineral is incorporated into ceruloplasmin, which distributes it to the tissues, allowing it to act as an essential cofactor in various enzymes.

Cu exhibits a variety of biological activities, notably its biocidal potential, which effectively prevents the growth of bacteria, fungi, and viruses [43]. Studies by Hosseini et al. [43] and Doremalen et al. [44] investigated the potential of Cu and Cu alloys against SARS-CoV-2 infection. These studies demonstrated that Cu has antiviral properties and is a promising candidate for combating the virus. Exposure of SARS-CoV-2 to Cu for four hours resulted in 2-log10 inactivation [43,44].

However, the breakdown of Cu homeostasis associated with deficiency or overload can have adverse consequences for human health, including low immunity, disruption of antioxidant species in the body, and the development of diseases such as diabetes and neurodegenerative disorders [45]. Several reports have recently highlighted the association between metabolic disorders and Cu immunosuppression. The most well-known of these is Menkes disease, which is characterized by systemic Cu deficiency [45]. In addition, Cu deficiency has also been linked to viral issues, as the supplementation of this metal is directly related to modulating immune responses through cytokine production and immune cell activity, including interleukin-2 (IL-2) production [46].

Cu deficiency impairs the production of pathogen-specific antibodies and defenses against infectious diseases. Studies evaluating the interaction between the Cu^2+^ complex and the S and N proteins of SARS-CoV-2 have demonstrated that ions can inhibit papain-like protease (PLpro), the enzyme responsible for the SARS-CoV-1 replication process. Thus, this trace element regulates not only the host’s immune responses, but also modifies the viral genome [42]. Cu’s ability to neutralise other infectious viruses, such as poliovirus, influenza A, and single- and double-stranded DNA and RNA viruses (whether enveloped or not), has also been demonstrated [47,48,49,50]. An extensive study of the Cu complex has revealed several mechanisms by which it combats HIV infection. These include stimulating the free radical-induced destruction of viral nucleic acids, blocking gp120 binding and viral fusion, increasing p24 synthesis and syncytium formation in HIV-infected lymphocytes, and persistently destroying the HIV protease enzyme. This enzyme is responsible for intracellular viral replication [47,51].

Hamlaoui et al. [50] synthesized two new Cu complexes (C-1 and C-2) named [(C_11_H_7_O_2_)(SCN)(C_10_H_8_N_2_)] and [(C_11_H_7_O_2_) (C_12_H_8_N_2_) Cl]·H_2_O, respectively. The ability of these complexes to inhibit the HIV-1 protease enzyme was evaluated using molecular docking. The results showed that the complexes occupied a common cavity in the receptor, suggesting that C-1 and C-2 block access to the active site of the HIV-1 protease. The authors also evaluated the binding energy, estimating it at −7.6 kcal/mol for C-1 and −8.3 kcal/mol for C-2. This suggests that the tested compounds form stable complexes when binding to HIV-1 protease. These results are considered promising with regard to the inhibitory effect of the designed complexes against the HIV-1 virus [50].

Influenza A (IAV) is a highly pathogenic, single-stranded RNA virus of zoonotic origin. It has caused several pandemics since the 1918 Spanish flu pandemic [52]. Lower respiratory tract infection is the leading cause of infectious disease-related mortality worldwide, and IAV infection generally exacerbates the production of ROS. This leads to the activation of signaling pathways sensitive to oxidative stress and induces apoptosis in respiratory epithelial cells [53,54]. To combat these species and protect the body against oxidative stress, ROS-catalyzing enzymes act to maintain balance, particularly SODs, for which Cu acts as a catalytic cofactor [55]. Consequently, Cu metabolism is closely related to changes in host cell metabolism, primarily via ceruloplasmin, a glycoprotein directly associated with reducing influenza virus reproduction during the initial and final stages of multiplication [55]. Therefore, maintaining the correct balance of Cu in the body is important for an effective immune response to IAV.

Kiseleva et al. [49] investigated the impact of Cu deficiency on IAV virus infection by conducting experiments on mice. Oxidase activity in the blood of the mice was experimentally reduced by the intraperitoneal injection of silver nanoparticles (AgNPs). The authors reported that treating the mice with AgNPs decreased the Cu concentration, as Ag atoms displaced Cu ions in the active centers of ceruloplasmin during the folding of this glycoprotein in the lumen of the Golgi complex. This reduced the activity of ceruloplasmin, decreasing the effectiveness of the immune response, which may lead to an increase in viral load.

Considering its relationship with the immune system, Cu immunosuppression is directly related to a greater viral effect on the human body. Conversely, excessive Cu accumulation can be toxic, particularly to mitochondria. To understand the effects of this overload of a trace element on the body, Tsvetkov et al. [55] identified a new form of cell death caused by excess Cu ions. This new form of cell death is called cuproptosis and has characteristics that differ from those of other known modes of cell death, such as apoptosis, autophagy, pyroptosis, and ferroptosis [56,57].

The cuproptosis mechanism begins with Cu entering the cell via ionophores or transporters. This causes Fe ions to migrate to the mitochondria via chaperone molecules. Then, Cu^2+^ is reduced to Cu by ferredoxin 1 (FDX1), which regulates lipoylation [58]. This reduction disrupts the function of iron–sulphur (Fe-S) clusters, which are essential for mitochondrial respiratory chain activity. The inhibition of these clusters is one of the main causes of Cu-induced toxicity, leading to the onset of cuproptosis. During this form of programmed cell death, Cu ions can bind to lipoylated enzymes, disrupting the tricarboxylic acid (TCA) cycle and promoting oligomerisation, thereby inducing proteotoxic stress [58]. Together with mitochondrial dysfunction, the interruption of the TCA cycle leads to an energy crisis and the activation of cell death pathways [58].

Cuproptosis is a relatively new form of cell death, and few studies have reported on its influence on viral infections. However, Hackler et al. [59] and Govind et al. [60] reported high levels of Cu in the bodies of survivors of the SARS-CoV-2 virus, and demonstrated that Cu and its alloys have enhanced antiviral properties, particularly against coronaviruses [59,60]. Wu et al. [57] built on these findings, hypothesising that the antiviral effects of Cu might be mediated by inducing cuproptosis in cells to facilitate the elimination of infected cells and the virus [57]. This suggests the possibility of developing targeted therapies through the cuproptosis pathway [56,61,62,63].

### 3.2. Role of Iron and the Immune System

Iron is another essential micronutrient for maintaining the quality of life of living organisms [64,65]. It is closely involved in fundamental biological processes, including the synthesis and metabolism of porphyrins and the regulation of the immune system [64,65]. This mineral is also essential for growth and defending the body against oxidative stress [65]. It is the most abundant trace element in the body, found mainly in the form of heme, with the highest concentrations found in hemoglobin (Hb). Macrophages in the spleen, liver, and bone marrow recycle this Fe when red blood cells undergo senescence. Fe circulates bound to transferrin, and any excess is stored primarily in hepatocytes within ferritin [66].

Excess Fe, also known as ferroptosis, is associated with bodily damage. This overload is caused by excessive abnormal hemolysis in the diet or hereditary disorders and is associated with liver damage. It triggers inflammation in the liver by activating nuclear factor kappa B (NF-κB), which promotes the release of pro-inflammatory cytokines and can lead to hepatic fibrosis [67]. Fe overload can result in the accumulation of free Fe, which promotes the generation of ROS through the Fenton reaction. This increases the body’s susceptibility to viral infections such as hepatitis C, HIV, cytomegalovirus, and SARS-CoV-2.

Hepatitis C is caused by an RNA virus belonging to the Flaviviridae family, which targets liver tissue, leading to cirrhosis and liver cancer [68]. HCV replicates within cells, leading to necrosis through immune-mediated cytolysis, hepatic steatosis, oxidative stress, and insulin resistance [67]. Recently, some studies have evaluated the effect of HCV infection on the recycling of the transferrin receptor (TfR1), which is an Fe-binding glycoprotein that has the potential to bind and release Fe [69]. In an in vitro test, HCV-infected cells exhibited decreased levels of *α*-xylin, a critical factor in TfR1 recycling. This resulted in reduced levels of active TfR1 protein and consequently increased intracellular Fe levels. Fe overload was observed in enterocytes, hepatocytes, and macrophages [69].

High levels of Fe influence the life cycle of HCV, affecting its replication and increasing reticulocyte levels in the form of ferritin (FTN). This trace element thus acts by weakening local antiviral defenses, causing accelerated fibrosis progression and increasing the risk of hepatocellular carcinoma [70]. Otha et al. [71] evaluated the molecular mechanisms contributing to increased loading in cells caused by HCV infection in mice. The authors identified two mechanisms of action: first, the initial transcriptional induction of hepcidin, the key hormone responsible for modulating iron homeostasis. HCV infection activates the cAMP-responsive element-binding protein hepatocyte-specific (CREBH) transcription factor, inducing the expression of bone morphogenetic protein 6 (BMP6). This results in an activated BMP-SMAD pathway that increases hepcidin promoter activity. The second mechanism involves post-translational regulation of the iron export membrane protein ferroportin 1 (FPN1), which is cleaved between residues Cys^284^ and Ala^285^ in the intracellular loop region of the central portion by HCV serine protease NS3-4A [71].

HIV is a single-stranded RNA enveloped retrovirus that uses the enzyme reverse transcriptase to convert its RNA into DNA and integrate itself into the genome of the host cell [72]. Recent studies have suggested that HIV replication is an Fe-dependent process as the virus triggers the release of hepcidin, an Fe-regulating hormone [73]. Deregulation of this hormone leads to higher levels of hepcidin in the body, which decreases Fe absorption and sequestration. This results in reduced Fe export from enterocytes to the blood, causing macrophages to accumulate in the spleen and liver [73]. However, increased concentrations of non-transferrin-bound iron (NTBI) from supplementation may be associated with an increased susceptibility to HIV, causing the infection to progress faster [74]. Studies have shown that high serum Fe levels are associated with increased oxidative stress in HIV-infected men and that Fe overload affects antiretroviral therapy (ART) due to the faster progression of HIV infection [75]. Chang et al. [75] conducted a study in which they altered the cellular iron levels of primary CD4^+^ T cells. The study monitored the relationship between Fe overload and HIV infection in serum samples taken from ten HIV-negative control patients and ten HIV-positive patients who were matched for age and race before antiretroviral therapy (ART). The authors reported that increased Fe concentration in the body is associated with increased susceptibility to HIV infection and replication, and that HIV infection influences cellular and systemic iron levels [75].

Human cytomegalovirus (HCMV) is a herpesvirus that causes disease in individuals with compromised or immature immune systems, such as transplant patients and neonates [76]. The virus adheres to cellular pathways to facilitate its own replication in infected cells and often modifies the cell’s protein composition to promote viral replication. Like the human immunodeficiency virus, HCMV depends on Fe for its cellular processes to function properly. In adequate amounts, Fe chelators can inhibit HIV-1 and HCMV infection [76]. However, an overload of this mineral has a different effect compared to HIV, facilitating the process of infection and viral replication [76]. This is because proteins containing Fe prosthetic groups are key to the biosynthesis and maturation of this virus, as well as to DNA replication and oxidative phosphorylation. Indeed, high Fe concentrations can promote the development of host cell cytomegaly and productive viral infection [76,77]. Xu et al. [78] evaluated the influence of long non-coding RNA encoded by HCMV (RNA2.7) on the maintenance of the ferroptosis inhibitor Fer-1, which is an Fe catalyst. The results revealed that RNA2.7 inhibits ferroptosis activity, thereby increasing ferritin and glutathione (GSH) levels. Therefore, iron accumulation aids host cell survival and complete viral replication [78].

SARS-CoV-2 infection is also an infectious disease that is highly influenced by Fe concentration. Studies report that high Fe levels can promote viral replication and that hyperferritinaemia in patients with SARS-CoV-2 infection has been associated with greater disease severity and hyperinflammation [79]. Hyperferremia results in elevated Fe levels and triggers a fulminant inflammatory response involving the acute release of pro-inflammatory cytokines, including IL6, TNF*α*, and CRP. This stimulates ferritin synthesis in patients with SARS-CoV-2 infection [80]. Excess Fe exacerbates the inflammatory process and stimulates oxidative stress by accumulating intracellularly in its free form [80,81]. This triggers erythrocyte dysfunction and the release of more heme Fe, which increases prothrombin time and D-dimer levels. These are cumulatively linked to severe coagulopathies that can lead to acute respiratory distress syndrome and multiple organ failure [81].

Gaiatto et al. [80] evaluated Fe, ferritin, and hepcidin levels, as well as transferrin receptor gene expression, in patients diagnosed with SARS-CoV-2. The study aimed to determine the profile of Fe metabolism and its relationship with the disease. The authors reported that SARS-CoV-2 interferes with the synthesis of proteins associated with Fe homeostasis. Additionally, altered Fe and ferritin levels were consistent with the inflammatory state of SARS-CoV-2 infection due to reduced hepcidin, the main regulator of Fe absorption. This corroborates the reduction in systemic iron absorption and the accumulation of iron in certain organs [80].

### 3.3. Role of Manganese and the Immune System

Manganese is an essential trace element involved in various biochemical and physiological processes. It acts as a cofactor for approximately 30 enzymes that are involved in different metabolic pathways, such as arginase, pyruvate carboxylase, glutamine synthetase, and glycosyltransferases [82]. In addition to its enzymatic functions, Mn contributes to cellular signalling processes. The element acts as a structural component in certain metalloproteins and modulates enzymatic activity, cell differentiation, organism development, and bone formation [83]. It also plays a decisive role in protecting the body against oxidative stress by acting as a cofactor of the enzyme manganese superoxide dismutase (MnSOD) in the mitochondria of cells [82,83]. Therefore, considering the recommended dietary allowance (RDA) for Mn in adults is 2.3 mg/day for men and 1.8 mg/day for women, dietary supplementation of this mineral is necessary [82].

Mn is predominantly absorbed in the small intestine, particularly in the duodenum and proximal jejunum. This process is mainly mediated by the DMT1 (Divalent Metal Transporter 1) transporter, which is also used by Fe. The efficiency of absorption is influenced by nutritional Fe status: it increases in cases of iron deficiency and decreases when there is an excess of Fe, Ca, P, or phytates in the diet [84]. Following absorption, Mn circulates in plasma bound to transferrin, albumin, and *α*_2_-macroglobulin, and is distributed throughout the tissues, particularly in the liver. The liver acts as the main regulator of metal homeostasis by promoting the excretion of excess manganese via the bile [84]. The average absorption of manganese in humans is relatively low, ranging from 3% to 5% of the total ingested amount [85]. However, certain conditions can significantly increase its uptake and absorption, highlighting the body’s strict control over this essential micronutrient.

A recent study showed that Mn can facilitate the maturation of dendritic cells (DCs) and macrophages, thereby enhancing the immune response to tumour-specific antigens via the cGAS-STING pathway [86]. This mechanism occurs when Mn is released from the mitochondria and the Golgi apparatus, causing it to accumulate in the cytosol, where it binds to cGAS. This increases cGAS sensitivity to double-stranded DNA (dsDNA) and its enzymatic activity. Mn also improves the binding affinity of cGAMP-STING and is considered a potent activator of cGAS, inducing cells to produce interferons (IFNs) and type I cytokines [87]. Furthermore, Mn can activate CD8 T cells and natural killer (NK) cells, thereby increase the number of memories CD8 T cells, as well as enhance innate and adaptive immune responses to tumors [88,89].

In another study, Lu et al. [90] reported that excess Mn reduces the levels of interleukins (IL)-2, -4, -6, -12*β*, and -17, as well as interferon (IFN)-*γ*, in chicken spleen lymphocytes. Excess Mn affects cytokine mRNA expression and causes immunosuppression in these cells [91]. Other studies have shown that an excess of this trace element impairs mitochondrial respiration and superoxide production in neutrophils, thereby reducing their ability to respond to infections [92].

### 3.4. Role of Selenium and the Immune System

Selenium is another essential trace element for the human body, known for its remarkable antioxidant and antimutagenic properties. It plays a fundamental role in metabolism and growth, and it acts in defense against microorganisms and parasites. In addition, Se has a crucial function in both the innate and adaptive immune systems and exhibits important anti-inflammatory effects [93].

The role of Se in the innate immune system involves maintaining physical barriers, supporting antimicrobial protein activity, enhancing cell motility, and promoting the phagocytic function of neutrophils and macrophages. It also regulates inflammation through its antioxidant action and cytokine modulation [93]. In adaptive immunity, Se is essential for the differentiation and proliferation of lymphocytes, as well as for antibody production and memory cell formation. Its deficiency impairs these functions, resulting in lower resistance to infections [94]. In terms of immunity, Se acts as a regulatory agent in the activation of T and B lymphocyte functions, as well as humoral immunity, potentially having a multifactorial impact [93].

Se deficiency is associated with increased oxidative stress and inflammation, which aggravates susceptibility and leads to poor outcomes in viral infections and respiratory diseases such as COVID-19 [95,96]. Ozdemir et al. [93] confirmed this relevance by showing that the vast majority of COVID-19 patients exhibited marked Se deficiency (87%) before treatment [94]. This finding reinforces that Se deficiency, by impairing antioxidant defense and immune response, contributes to an unfavorable prognosis, highlighting that correcting this micronutrient deficiency may support recovery and balance immune responses in COVID-19 [97].

During viral infections, there is a significant increase in the production of ROS, leading to an imbalance between their generation and the endogenous antioxidant systems, resulting in oxidative stress [98]. This altered redox state promotes damage to proteins, lipids, and nucleic acids, and activates inflammatory pathways such as NF-κB, stimulating the release of pro-inflammatory cytokines, including TNF-α and IL-6 [99,100]. Se, in turn, plays a central role in maintaining redox homeostasis through its incorporation as selenocysteine into selenoproteins, such as glutathione peroxidases (GPXs) and thioredoxin reductases (TXNRDs) [101]. These enzymes are involved in the neutralization of hydroperoxides, the regeneration of intracellular antioxidants, and the activation of the Nrf2 pathway, which is essential for antioxidant and anti-inflammatory defense. Se deficiency reduces the activity of these selenoproteins, impairing immune responses and favoring viral replication, increased oxidative stress, and viral mutagenesis, thereby enhancing the virulence of several pathogens [102,103]. Despite its known immune benefits, there is still limited information on the direct impact of vitamin E or Se supplementation in humans with COVID-19, although it is recommended that patients maintain adequate intake of these antioxidant nutrients.

The main effects of Se deficiency and supplementation on oxidative and immunological mechanisms during viral infections are summarized in Table 1.

Table 1 shows that viral genomic alterations and impairment of the host immune response are the main mechanisms associated with Se deficiency, as they influence both the susceptibility to and the progression of viral infections [114,115,116,117,118,119,120]. In this context, strategically, it is crucial to adopt a personalized approach by assessing the patient’s baseline Se status before supplementation [118].

### 3.5. Role of Zinc and the Immune System

Zinc is an essential trace element, present in the body in amounts of around 2 to 3 g, predominantly in its intracellular form. It acts as a cofactor and structural component of various enzymes, performing crucial functions in cell signaling and immune regulation. Its action is fundamental for the activation, differentiation, and function of defense cells, such as T and B lymphocytes, neutrophils, macrophages, NK cells, and dendritic cells. Additionally, Zn preserves the integrity of epithelial barriers and activates antioxidant enzymes, such as superoxide dismutase, protecting cells against oxidative damage caused by ROS [121,122,123]. Table 2 summarizes the main Zn function in the biological system.

In general, Zn deficiency impairs the function of immune cells, the integrity of epithelial barriers, and the regulation of inflammatory responses, increasing susceptibility to infections [121,122,130]. In contrast, excessive Zn intake can be toxic and may reduce immune cell function in a manner similar to Zn deficiency, showing that both excess and deficiency are harmful. High Zn levels can stimulate the dysregulated production of inflammatory cytokines, promoting an excessive immune response [131,132,133]. Therefore, when Zn levels are adequate and balanced, the immune system enhances the body’s defense response, increasing immune cell count and strengthening natural immunity.

In viral infection, Zn acts in defense against respiratory viral infections by strengthening mucociliary barriers and enhancing the action of antimicrobial peptides, such as lysozymes and interferons [134,135]. It strengthens innate immunity by stimulating mucociliary clearance, increasing ciliary beat frequency, and preserving the integrity of the respiratory epithelium by maintaining tight junction proteins such as Claudin-1 and ZO-1, in addition to inhibiting apoptosis. Additionally, Zn modulates viral entry mechanisms by interfering with the expression and structure of angiotensin-converting enzyme 2 (ACE2), a zinc-dependent metalloenzyme, possibly reducing its affinity for the virus through conformational effects and regulation via Sirt-1 [136]. Another relevant effect is the ability to restore the production and signaling of interferons, essential for the antiviral response, via the JAK/STAT pathway, as well as exerting anti-inflammatory action by inhibiting LFA-1/ICAM-1 interaction, which reduces leukocyte recruitment to the lung [137].

Moreover, Zn can prevent viruses such as coronaviruses from replicating, which may explain its potential protective and therapeutic effects in viral infections [95]. Maintaining adequate Zn levels is crucial, as studies have shown that population Zn status is directly associated with the prevalence of respiratory infections in both children and adults [95,138]. In vitro assays have demonstrated that Zn exhibits antiviral activity by inhibiting SARS-CoV RNA polymerase and has been suggested as an immune support and prophylactic agent against H1N1 influenza (“swine flu”) [96], HCV [139,140,141,142], HEV [143,144,145], HAV [146], and HBV [147,148]. In this context, Zn plays a crucial role in protecting the body against viral infections, by maintaining a strong and well-balanced immune system [128,149].

### 3.6. Interactions Among Trace Elements in Immune Homeostasis

Although the individual roles of zinc, copper, and selenium have been discussed separately, growing evidence indicates that these micronutrients act as a coordinated network rather than independent regulators of immune function. Their absorption, distribution, and biological activities are strongly interconnected, and imbalances in one element frequently affect the homeostasis of the others.

**Figure 3:**
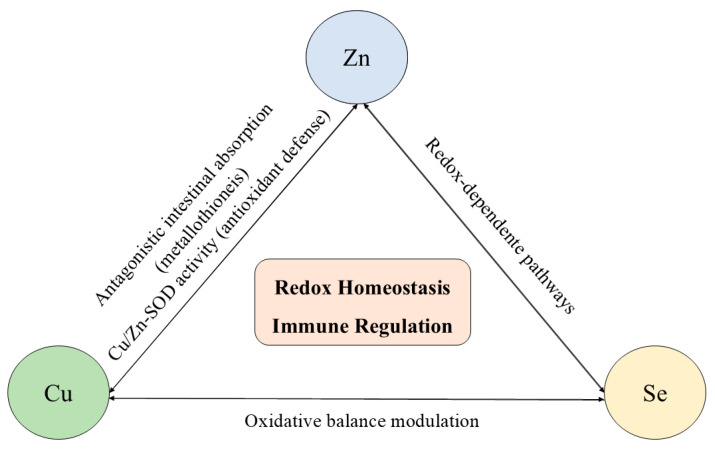
Interactions among zinc (Zn), copper (Cu), and selenium (Se) in redox homeostasis and immune regulation. These trace elements exhibit antagonistic and cooperative interactions that regulate intestinal absorption, enzymatic activity, and oxidative balance. Zinc and copper interact antagonistically through metallothionein-mediated absorption and jointly participate in Cu/Zn-superoxide dismutase activity. Selenium modulates redox-sensitive pathways that influence Zn- and Cu-dependent enzymes and immune signaling. Disruption of this coordinated network, either by deficiency or excess, results in redox imbalance and impaired immune responses.

In addition to their individual functions, Zn, Cu, and Se exhibit strong interdependent interactions in absorption, distribution, and biological activity, which are particularly evident in conditions of simultaneous deficiency. Experimental evidence shows that combined deficiencies disrupt tissue distribution patterns, leading to compensatory accumulation or depletion of these elements in specific organs, thereby amplifying metabolic imbalance and immune dysfunction. Experimental studies demonstrate that combined deficiencies do not result in uniform depletion but induce compensatory and, in some cases, maladaptive redistribution of these trace elements by organs such as the liver, spleen, kidneys, and intestine [150]. The integrated interactions among these trace elements are illustrated in Figure 3.

Zinc and copper share common intestinal transport mechanisms, and excessive intake of one can competitively inhibit the absorption of the other, often resulting in secondary deficiencies. High zinc intake induces the expression of metallothionein, which preferentially binds to copper in enterocytes, reducing its systemic availability and potentially impairing immune cell maturation and antioxidant defense. Selenium further modulates this balance, as selenoproteins interact functionally with Cu- and Zn-dependent enzymes, such as superoxide dismutase and ceruloplasmin, to maintain redox homeostasis [150,151,152,153,154,155].

It is important to note that both deficiency and excess of these trace elements can be harmful. While insufficient levels compromise antioxidant capacity, immune signaling, and cellular integrity, overload, particularly of copper and zinc, can promote oxidative stress, mitochondrial dysfunction, and immunotoxic effects [150,155,156]. These findings emphasize that immune competence depends not only on adequate intake of individual micronutrients but also on their balanced interaction, highlighting the need for cautious supplementation strategies based on nutritional status rather than isolated micronutrient replacement.

### 3.7. Other Elements and the Immune System

In addition to the trace elements already discussed, other trace elements play important roles in immune regulation and resistance. For instance, the trivalent chromium (Cr^3+^) participates in the metabolism of insulin, lipids, and proteins, contributing to glycemic homeostasis and modulation of immune function [157]. Although its deficiency is rare, alterations in its levels can affect immune response and oxidative balance. On the other hand, high doses of Cr^3+^ supplements may cause hepatic and renal toxicity, highlighting the importance of controlling its intake [157].

Molybdenum (Mo) acts as a cofactor for redox enzymes, including xanthine oxidase and sulfite oxidase, which are essential for antioxidant defense and cellular detoxification [158]. Although Mo deficiency is rare in the general population due to its widespread dietary availability, it has been reported under conditions such as long-term parenteral nutrition or severe metabolic disorders. Conversely, excessive Mo intake may disrupt trace metal homeostasis, particularly by antagonizing Cu absorption and bioavailability. This interaction can lead to secondary Cu deficiency, impairing the activity of Cu-dependent enzymes such as cytochrome c oxidase and SOD [158]. From an immunological perspective, Cu deficiency induced by elevated Mo levels can compromise innate immune responses by impairing macrophage and neutrophil function, including reduced phagocytic activity, diminished respiratory burst, and altered cytokine production. These effects collectively weaken host defense mechanisms against pathogens and may increase susceptibility to infections [158]. Therefore, maintaining Mo homeostasis is crucial not only for metabolic and detoxification pathways but also for preserving immune competence through its intricate interplay with other essential trace elements [158].

Iodine (I) is essential for the synthesis of thyroid hormones (T3 and T4), which control metabolism and directly influence immune cell differentiation and proliferation [159,160]. I deficiency results in hypothyroidism and compensatory thyroid enlargement (goiter). These endocrine disturbances are associated with impaired immune competence, particularly affecting adaptive immunity and antibody production [161]. Reduced thyroid hormone levels have been linked to altered T-cell differentiation, diminished B-cell activation, and decreased antibody production, ultimately compromising humoral immune responses and increasing susceptibility to infections [161]. Conversely, excessive I intake can disrupt thyroid homeostasis and immune tolerance, potentially triggering or exacerbating autoimmune thyroid diseases such as Hashimoto’s thyroiditis [162]. High iodine levels may promote increased antigenicity of thyroglobulin and enhance oxidative stress within thyrocytes, leading to aberrant activation of autoreactive T and B cells. This immune dysregulation results in chronic inflammation and progressive destruction of thyroid tissue, underscoring the narrow physiological window between sufficiency and toxicity [162]. In this scenery, these findings highlight the critical role of iodine homeostasis in maintaining endocrine–immune crosstalk, where both deficiency and excess can negatively impact immune regulation and thyroid health.

Cobalt also plays a fundamental physiological role. It acts as a component of vitamin B_12_, participates in the synthesis of various enzymes, and is involved in metabolic processes and the stimulation of hematopoiesis. Controlled Co release in the body can activate the immune system, offering therapeutic strategies against infectious diseases and contributing to the strengthening of immune responses. Based on these properties, Co nanoparticles (CoNPs) have been extensively investigated as promising therapeutic agents, capable of inducing the production of ROS, which are associated with their inhibitory effects on different types of bacteria, fungi, and viruses [163,164]. Mechanistically, CoNPs and hybrid materials can directly interact with viral proteins and structural components, promoting viral inactivation through binding and van der Waals forces [165], releasing Co ions that disrupt critical viral functions, and inducing ROS generation that damages essential viral and cellular components [166]. Moreover, when incorporated into matrices or doped systems (e.g., Co-doped ZnO), these materials enhance antiviral activity and enable combined strategies (viral inactivation + therapeutic delivery) [167]. As drug carriers, functionalized Co ferrite nanoparticles have shown potential for targeted delivery and sustained release of antiviral agents within cellular reservoirs, thereby enhancing the efficacy of antiretroviral therapies [168]. In the vaccine field, CoNPs act as antigen encapsulation/conjugation platforms, increasing antigen persistence, recruitment and activation of antigen-presenting cells, and the induction of adaptive immune responses, features desirable for the development of novel vaccine adjuvants or delivery vectors [169,170,171,172].

Finally, lead (Pb) stands out as a toxic element with no known biological function but significant immunosuppressive effects. Sepehri et al. [173] reported that elevated Pb levels in tuberculosis patients impair antibody production, reduce lymphocytic activity, and compromise phagocytic response, thereby increasing susceptibility to infections. Chronic lead exposure is associated with dysregulation of adaptive immunity, persistent oxidative stress, and a higher risk of autoimmune diseases [174].

In this context, it is observed that both deficiency and excess of trace elements, whether essential or toxic, can compromise immune response effectiveness, exacerbate inflammatory processes, and alter the course of viral infections. Maintaining adequate levels of these micronutrients is therefore crucial to sustain immune and metabolic integrity, representing a key factor in the prevention and management of infectious diseases.

## 4. Trace Elements as Therapeutic Potential Against Viral Infections

As described in the previous sections, trace elements play a fundamental role in maintaining human health, particularly in supporting a robust immune response against viral pathogens [175,176]. During the course of a viral infection, the physiological demand for these micronutrients increases significantly, reflecting their contribution to regulating immune system functions [177]. This includes the proliferation and activation of lymphocytes, as well as increased antioxidant defense mechanisms mediated by neutrophils and macrophages [1]. The immunopathology of viral infections often involves complex interactions between host immunity and oxidative stress pathways. In this context, several trace elements, including Cu, Fe, Mn, Se, and Zn, are dynamically modulated during the infectious process. These elements are essential for a wide range of cellular and molecular processes, including enzymatic antioxidant defense (e.g., superoxide dismutase, glutathione peroxidase), cytokine signaling, and the regulation of inflammatory mediators [1,177].

However, imbalances in trace element homeostasis during infection can increase disease progression [12]. For example, deficiencies or excesses can contribute to immunosuppression, impaired leukocyte function, and increased oxidative stress, ultimately compromising the host’s ability to effectively eliminate the virus. In some cases, viral pathogens can even manipulate the host’s trace element metabolism to favor their replication [12].

Given these factors, maintaining adequate levels of trace elements, whether through dietary intake, supplementation, or therapeutic modulation, has represented an important strategy in the treatment of viral infections. Evidence suggests that targeted interventions to restore trace element balance can increase immune competence, reduce viral load, and mitigate oxidative tissue damage, thus improving clinical outcomes [178]. For instance, Asdamongkol et al. [179] investigated the immunomodulatory effects of Zn supplementation in HIV-infected individuals with immunological discordance. This condition was characterized by patients on antiretroviral therapy (ART) who maintained suppressed viral loads, but failed to achieve sufficient immunological recovery, defined as a CD4^+^ T-cell count ≤ 200 cells/mm^3^ and a less than 30% increase from baseline after 12 months of virological suppression. Among the 31 participants, 12 individuals had plasma Zn deficiency. Of these, 5 patients with low plasma Zn levels and 8 with normal Zn levels were randomly assigned to receive oral Zn supplementation. After the intervention, the median increase in plasma Zn concentration among deficient individuals was 29 µg/dL, compared with only 4.5 µg/dL in the placebo group. Notably, patients with initially low Zn levels showed a statistically significant improvement in CD4^+^ T-cell counts after supplementation. These findings suggest that Zn supplementation may improve immunological recovery in HIV-positive patients with discordant responses to ART [179]. However, larger, long-term clinical trials are needed to evaluate the sustained immunological and clinical benefits of Zn supplementation in this subgroup.

In another study, Hurwitz et al. [180] examined the potential of Se supplementation to reduce HIV-1 viral load. The study used a double-blind, randomized, placebo-controlled design, in which participants received a daily dose of 200 µg of Se-rich yeast. After a 9-month treatment period, the results revealed a correlation between the serum response to Se and clinical outcomes. Participants in the Se group who experienced minimal increases in serum Se levels demonstrated lower adherence, increased HIV-1 viral load, and a decline in CD4^+^ T-cell count. In contrast, those with more substantial increases in serum Se levels had significantly better adherence, stable viral load, and increased CD4^+^ T-cell count. These findings suggest that consistent Se supplementation may contribute to the stabilization of viral replication and support immune function in individuals living with HIV-1 [180]. The data reinforce the potential role of Se as a low-cost and well-tolerated adjunctive therapy within the broader framework of HIV clinical treatment.

The properties of Cu against viral infection have also been extensively investigated [181]. For instance, Nakano et al. [181] evaluated the effect of Cu against SARS-CoV-2 infection. In this study, purified viral particles were used to specifically evaluate the mechanisms of viral inactivation and identify structural damage induced by Cu exposure. The results demonstrated a substantial reduction in viral infectivity within 30 min. Furthermore, a significant decrease in spike protein levels in the viral envelope was detected using enzyme-linked immunosorbent assay (ELISA). The results suggest that the combined action of Cu(I) and Cu(II), along with reactive oxygen species such as superoxide, plays a critical role in the disruption and inactivation of SARS-CoV-2. These results highlight the potential of Cu as an effective antiviral agent [181].

During viral infections, trace element homeostasis is frequently altered, leading to redistribution and changes in serum concentrations that may impair immunocompetence. Deficiencies in key elements such as zinc and selenium are associated with reduced immune efficiency, whereas adequate micronutrient status supports both innate and adaptive immune responses and may positively influence the course of viral diseases [21].

In contrast to the extensively studied roles of Zn, Se, Cu, and Fe, other trace elements such as cobalt (Co), molybdenum (Mo), and iodine (I) have received comparatively less attention in the context of viral infections. Nevertheless, emerging evidence suggests that these elements may also influence host–virus interactions through immunomodulatory, antiviral, and redox-related mechanisms [21,182]. Molybdenum acts as a cofactor for key oxidoreductases, including xanthine oxidase and sulfite oxidase, which are involved in redox homeostasis and immune cell metabolism [183]. Although direct antiviral effects of molybdenum supplementation remain insufficiently characterized in clinical settings, preclinical studies indicate that molybdenum-containing compounds can inhibit viral polymerases, particularly in HIV-related models, highlighting a potential, yet largely experimental, antiviral role [184].

Similarly, cobalt plays an essential biological role, primarily as a constituent of vitamin B12, which is required for DNA synthesis, erythropoiesis, and immune cell proliferation [185]. Beyond its nutritional relevance, cobalt-based complexes, such as the Co(III) Schiff base compound doxovir, have demonstrated antiviral activity against drug-resistant herpes simplex virus strains, reaching clinical evaluation for topical use [186,187,188]. However, it is important to distinguish these pharmacological cobalt complexes from dietary cobalt supplementation, as evidence supporting systemic antiviral benefits from cobalt intake alone remains limited and largely indirect [189,190].

Iodine represents a distinct case among trace elements, as its antiviral relevance is primarily associated with its potent antimicrobial properties rather than systemic supplementation. Molecular iodine and iodine-based formulations exhibit broad-spectrum virucidal activity through oxidative damage to viral proteins and nucleic acids and are widely used as topical antiseptics [191,192]. While I is essential for thyroid hormone synthesis and immune regulation, evidence supporting oral iodine supplementation as a strategy to enhance antiviral immunity remains limited and highly context-dependent [193,194,195].

Table 3 provides an overview of the main trace elements used as therapeutic strategies against viral infections, highlighting their recommended daily intake, bioavailability, potential toxicity and primary dietary sources.

From a nutritional standpoint, adequate intake of these trace elements is guided by specific reference values for each age group, which differ substantially from any pharmacological applications. For molybdenum, recommendations range from adequate intakes of 2–3 µg/day in infants to 45 µg/day in adults, increasing to 50 µg/day during pregnancy and lactation, with a tolerable upper limit set at 2000 µg/day [195]. Iodine shows greater age variability, with 110–130 µg/day in infancy, 90–120 µg/day in children, 150 µg/day from adolescence onwards, and high requirements during pregnancy and lactation (220–290 µg/day), while the upper limit for adults is 1100 µg/day, above which the risk of thyroid dysfunction increases [196]. For cobalt, there is no independent RDA, since its biological function is restricted to the structure of vitamin B12, for which the recommendation is approximately 2.4 µg/day for adults. It is important to note that these values are intended exclusively for physiological maintenance in healthy individuals and should not be interpreted as therapeutic doses [197]. Among the elements discussed, only iodine has an established clinical application in supraphysiological doses, in the form of radioiodine (I-131) for the treatment of thyroid disorders, while there are no recognized therapeutic protocols for molybdenum or cobalt, and excessive exposure can result in systemic toxicity [198].

Based on these studies, it is possible to observe that the presence of a viral infection can cause not only a systemic redistribution of these elements, but also a significant depletion of their reserves, compromising the effectiveness of the immune response [24] and justifying the growing attention to the role of trace element supplementation as an adjuvant therapeutic strategy in modulating immunity and mitigating damage associated with viral progression [24]. However, they reinforce the need for a personalized medicine approach to trace element supplementation in viral infections. Baseline assessment of micronutrient status, disease severity, comorbidities, age, and nutritional history should guide supplementation strategies [190]. Instead of universal administration, trace elements should be used in a contextualized manner, targeting deficient or high-risk subgroups, carefully monitoring the dose, duration, and potential adverse effects. This approach maximizes therapeutic benefit and minimizes unintended harm [174,182].

## 5. Conclusions

Viruses are infectious agents that are among the leading causes of human disease, producing acute and chronic illnesses ranging from mild infections to systemic syndromes, including human immunodeficiency virus (HIV), hepatitis B and C (HBV, HCV), SARS-CoV-2, and influenza, among others. Trace elements play fundamental roles in regulating the immune response and controlling viral replication, demonstrating a dual role as protective modulators and facilitators of infection. The relationship between trace elements and viral infections is complex, as the specific functions of various elements remain undefined. So, further research is needed to elucidate the specific effects of each trace element on different viral infections, enabling the investigation of approaches that enhance antiviral immunity without promoting viral replication, thus contributing to integrated strategies for the prevention and treatment of viral diseases. In this regard, randomized pilot clinical trials may be conducted to test targeted micronutrient interventions, considering more personalized and stratified supplementation with rigorous toxicity assessments in order to provide valuable data to support advances in the field of nutritional immunovirology.

## Figures and Tables

**Table 1:** Main effects of selenium (Se) deficiency and supplementation on oxidative and immunological mechanisms in viral infections.

Virus (Type)	Mechanism	Effects	Supplementation	Ref.
Coxsackie B (RNA, *Picornaviridae*)	Increased oxidative stress and viral mutation; reduced GPX and TXNRD activity	Myocarditis (Keshan disease), increased virulence	Disease prevention and restoration of antioxidant activity	[103,104,105]
Influenza A (RNA, *Orthomyxoviridae*)	Alteration of redox balance and adaptive immune response	Increased viral replication and pulmonary inflammation	Reduction in inflammatory cytokines (IL-6, TNF-α) and improvement of immune response (selenium nanoparticles)	[103,106,107]
HIV (RNA, *Retroviridae*)	Depletion of GPX and GSH; increased oxidative stress and immune damage	Decrease in CD4 lymphocytes and disease progression	Maintaining CD4 levels and reducing viral load	[103,108,109]
Hepatitis B/C (DNA/RNA, *Hepadnaviridae/Flaviviridae*)	Hepatic redox dysfunction and increased ROS	Liver damage and increased risk of hepatocellular carcinoma	Reduction in liver inflammation and progression to HCC	[103,110,111]
Poliovirus (RNA, *Picornaviridae*)	Increased oxidative stress and viral mutagenesis	Reduced vaccine response and increased replication	Improved immune response and vaccine protection	[103,112]
SARS-CoV-2 (RNA, *Coronaviridae*)	Exacerbation of oxidative stress and activation of NF-κB	Greater severity of COVID-19 and systemic inflammation	Improved antioxidant defense and lower mortality	[103,113]

**Table 2:** Mechanisms of zinc action and their immunological and biochemical relevance.

Mechanism	Function	References
Signaling and regulation	Ion signal, regulation for transporters, vesicles, and metals	[124]
Catalytic, structural, and regulatory function	Enzyme cofactor, protein and membrane stabilization	[125,126]
Modulation of transcription factors	Influence on factors such as NF-kB, AP-1, zinc finger domains	[126]
Cytokine regulation	Influence on the production and signaling of inflammatory cytokines	[126,127]
Innate and adaptive immunity	Development and function of neutrophils, NK cells, macrophages, T cells, and B cells	[36,127,128]
Hormone and lymphocyte activation	Activation of thymic hormones (thymulin) to stimulate T lymphocytes	[129]
Antioxidant protection	Reduction in oxidative damage and stabilization of membranes	[36,126,129]
Regulation of apoptosis	Influence on programmed cell death and gene expression	[37,129]
Zinc deficiency/excess	Immunosuppression or immune dysfunction	[37]

**Table 3:** Information on key trace elements used in supplementation as adjuvant therapy against viral infections.

Element	Recommended Daily Allowance (RDA) *	Factors Affecting Absorption (Bioavailability)	Toxicity Risk	Main Food Sources
Co	There is no specific recommended intake for isolated Co in many countries, as it is part of vitamin B12; as a free trace element [182]	Bioavailability generally via foods with vitamin B12 or ionic forms; organic compounds are better absorbed [182]	Excessive exposure can cause toxic effects on the cardiovascular, thyroid, pulmonary and dermatological systems, depending on the chemical form, while the margin between essential intake and toxicity remains relatively narrow [182]	Food rich in vitamin B12 (which contains Cot as part of the molecule), seafood, meat, liver, some vegetables depend on soil [182]
Cr	Approximately 35 µg for adults (USA) for Cr^3+^ (dietary extracts) [183]	Low to moderate bioavailability; trivalent (Cr^3+^) forms essential; hexavalent (Cr^6+^) toxic; interactions with other nutrients; chemical form is important [183]	Toxicity of industrial forms, Cr^6+^: carcinogenic, renal/lung toxic; for Cr^3+^ via diet, toxicity rarely documented [183]	Meat, poultry, fish, beer, whole grains, fruits, vegetables (depending on soil), spices [183]
Cu	Approximately 0.9 mg for adults (men and women) in the USA [184]	Absorption depends on form (organic, chelated, or inorganic), dietary Zn and Fe levels (competition), intestinal integrity; ceruloplasmin helps with metabolism [185]	Possible toxicity at very high ingestions (10 mg/day): gastrointestinal symptoms, after high exposures there may be liver/kidney damage [186]	Liver, shellfish, nuts, seeds, whole grains, dark chocolate, cocoa products, legumes [184]
Fe	Approximately 8 mg for men and 18 mg for women of reproductive age in the USA [187]	High heme absorption (meat); lower non-heme absorption (vegetables, grains); vitamin C increases absorption; phytates, polyphenols reduce absorption [187]	Excess can cause overload, toxicity, free Fe raises oxidative stress, risk of diseases such as hemochromatosis [187]	Red meat, liver, poultry, fish; legumes, dark leafy vegetables, fortified cereals [187]
Mn	Approximately 2.3 mg for adult men, and 1.8–2.0 mg for women in USA [188]	Moderate absorption; competitions with Fe and Ca [188]	Toxicity at high exposures (~11 mg/day). It can cause neurotoxicity and neurological symptoms [188]	Nuts, tea, legumes (beans, peas), whole grains, some dark vegetables [188]
Mo	Approximately 45 µg for healthy adults (≥19 years) in USA [189]	Very well absorbed (generally 70–90% from food forms), although certain foods such as tea may reduce absorption. Prepared forms or supplements tend to be more absorbed [189]	Generally, mildly toxic (~2 mg/day for adults). Rare adverse effects seen at very high doses or in animals. Possible interaction with Cu under specific conditions [189]	Legumes, grains, nuts, organ meats, soybeans, vegetables (kale, etc.) [187]
Se	Approximately 55 µg for adults (men and women) in USA [190]	High, especially in organic forms (selenomethionine, found in foods such as Brazil nuts). Absorption ranges from 70% to 90%. Inorganic forms (selenite, selenate) are also well absorbed, but have different metabolisms [190]	Excess (400 µg/day) can cause selenosis, characterized by symptoms such as hair loss, nail changes, nausea, fatigue, and neurological changes. Toxicity is rare from food, but possible from high-dose supplements [190]	Brazil nuts (very high concentration), seafood (tuna, sardines), beef liver, eggs, whole grains, brown rice, mushrooms. The content depends largely on the soil where the food was grown or raised [190]
Zn	Approximately 11 mg for men and 8 mg women adults in USA [191]	Absorption depends on form (hemic or food), presence of phytates (in grains/pulses) reduces absorption. It can interact with Cu and Fe [191]	Excess toxicity (>40 mg/day) can lead to Cu deficiency, gastrointestinal disturbances, and effects on the immune system [191]	Oysters, red meat, poultry, seafood, dairy, fortified cereals, legumes [191]

* RDA = Recommended Dietary Allowance (healthy adults).

## Data Availability

No new data were created or analyzed in this study. Data sharing is not applicable to this article.

## Abbreviations

ACE2	Angiotensin-converting enzyme 2
AgNPs	Silver nanoparticles
Ala	Alanine
ART	Antiretroviral therapy
AP-1	Activator protein 1
BMP6	Bone morphogenetic protein 6
CCL4	CC motif ligand 4
CD4	Cluster of differentiation 4
cGAS	Cyclic GMP–AMP synthase
COVID-19	Coronavirus disease 2019
CREBH	Cyclic AMP responsive element-binding protein H
Ctr1 transporter	Copper transporter 1
Cu-Zn SOD	Copper–zinc superoxide dismutase
CVB3	Coxsackievirus B3
CXCL1	CXC motif Ligand 1
DAAs	Direct-acting antivirals
DCs	Dendritic cells
DMT1	Divalent metal transporter 1
DNA	Deoxyribonucleic acid
DsDNA	Double-stranded deoxyribonucleic acid
ELISA	enzyme-linked immunosorbent assay
FDX1	Ferredoxin 1
Fe-S	Iron-sulphur
FPN1	Ferroportin 1
FTN	Ferritin
GMP-AMP	Guanosine monophosphate–adenosine monophosphate
GPXs	Glutathione peroxidases
GSH	Glutathione
gp120	Glycoprotein 120
H1N1	Hemagglutinin type 1 and neuraminidase type 1
HAV	Hepatitis A Virus
Hb	Haemoglobin
HBC	Hepatitis C virus
HBV	Hepatitis B virus
HCC	Hepatocellular carcinoma
HCMV	Human cytomegalovirus
HIV	Human immunodeficiency virus type 1
HSV	Herpes simplex virus
IAV	Influenza A
IFNs	Interferons
IL-1β	Interleukin-1 beta
IL-2	Interleukin-2
IL-6	Interleukin-6
IL-8	Interleukin-8
K	Potassium
LFA-1/ICAM-1	Lymphocyte function-associated antigen 1/Intercellular adhesion molecule 1
LOX	Lysyl oxidase
MnSOD	Manganese superoxide dismutase
NF-κB	Nuclear factor kappa B
NK	Natural killer
Nrf2	Nuclear factor erythroid 2–related factor 2
NTBI	Non-transferrin-bound iron
p24	Protein 24
PLpro	Papain-like protease
RDA	Recommended dietary allowance
RdRp	RNA-dependent RNA polymerase
RNA	Ribonucleic acid
RNS	Nitrogen species
ROS	Reactive oxygen species
SARS-CoV-2	Severe acute respiratory syndrome coronavirus 2
SeNPs	Selenium nanoparticles
SOD	Superoxide dismutase
SODs	Superoxide dismutases
TCA	Tricarboxylic acid
TfR1	Transferrin receptor
TNF-α	Tumor necrosis factor alpha
TXNRDs	Thioredoxin reductases
ZO-1	Zonula occludens-1
